# Supplementary material for: Fuel trait effects on flammability of native and invasive alien shrubs in coastal fynbos and thicket (Cape Floristic Region)
Source: PeerJ. 2022 Jul 28;10:e13765. doi: 10.7717/peerj.13765 (PMC9339215; doi:10.7717/peerj.13765)
Supplement: Supplemental Information 1 — The particular flammability measure studied is specified, i.e., ignitability (expressed as ignition frequency, or the inverse, time to ignition), combustibility (expressed as maximum temperature, heat released, flame height, rate of burn, or rate of spread), sustainability (expressed as burn duration), and consumability (expressed as completeness of burn, or proportion of biomass consumed). Fuel traits considered were fine fuels, coarse fuels, dead fuels, fuel load (dry biomass quantity, or measures of bulk density), fuel bed porosity (the inverse being fuel density or packing ratio), and fuel moisture (the inverse being plant dry matter content). ‘+’ denotes a positive effect, ‘−’ a negative effect, and ‘0’ no effect of fuel traits on flammability. [file peerj-10-13765-s001.docx]

**SUPPLEMENTARY 1**

Flammability studies conducted in vegetation types that sustain grass sward- or canopy level fires (as opposed to dry litter beds), with an indication of the scale of the experiment (i.e. leaves, plant shoots or field plots). The particular flammability measure studied is specified, i.e. ignitability (expressed as ignition frequency, or the inverse, time to ignition), combustibility (expressed as maximum temperature, heat released, flame height, rate of burn, or rate of spread), sustainability (expressed as burn duration), and consumability (expressed as completeness of burn, or proportion of biomass consumed). Fuel traits considered were fine fuels, coarse fuels, dead fuels, fuel load (dry biomass quantity, or measures of bulk density), fuel bed porosity (the inverse being fuel density or packing ratio), and fuel moisture (the inverse being plant dry matter content). ‘+’ denotes a positive effect, ‘-’ a negative effect, and ‘0’ no effect of fuel traits on flammability.

| **Source** | **Study species, vegetation type; location** | **Scale of experiment** | **Flammability measure** | **Fuel traits** | | | | | | **Notes** |
| --- | --- | --- | --- | --- | --- | --- | --- | --- | --- | --- |
|  |  |  |  | **Fine** | **Coarse** | **Load** | **Porosity** | **Dead** | **Moisture** |  |
| Alam et al., 2019 | 43 species of various growth forms and vegetation types; New Zealand | Leaves and plant shoots | Ignitability  Maximum temperature  Sustainability  Consumability |  | -  - |  |  |  | -  -  -  - | Leaf thickness negatively affected ignitability.  Phenolics content increased maximum temperature, while lignin content increased maximum temperature, burn time, and completeness of burn. Phosphorous and cellulose contents did not affect flammability.  Overall, leaf traits explained substantial variation in shoot flammability. |
| Alessio et al., 2008 | 4 species from Mediterranean shrubland; Mediterranean Basin, Spain | Leaves | Flammability (largely ignitability) |  |  |  |  |  | - | Flammability depended on leaf moisture content within all species, with lesser effects of volatile terpene contents. |
| Burger and Bond, 2015 | 29 species from Cape shrublands; South Africa | Plant shoots | Flammability  Consumability | +  + |  |  | 0 | + | 0 | Maximum temperature positively correlated with % fuel burned.  Moisture content was not significant but accounted for plant flammability to some extent. |
| Calitz et al., 2015 | 99 species from five biomes, Fynbos, Grassland, Nama-Karoo, Thicket, Forest; South Africa | Plant shoots | Flammability | + | - | + | - |  | - | General lack of robust relationships between fuel traits and flammability but trends which confirm other studies’ findings. |
| Cubino et al., 2018 | 51 native and alien species of various growth forms from tussock grasslands; South Island, New Zealand | Plant shoots and whole plants | Ignitability  Combustibility  Sustainability  Consumability |  | + | +  + | 0  0  0  0 |  | 0  0  0  0 | Total fresh biomass was the key shoot trait affecting flammability.  This study assessed shoot and whole plant flammability and upscaled that to community flammability by modelling flammability based on fuel traits and species abundance. |
| Davies and Legg, 2011 | *Calluna vulgaris* dominated moorland; United Kingdom | Plots  (2m x 2m) | Ignitability  Rate of spread |  |  |  | - | + | -  - | Ignition and initial rate of fire spread was primarily controlled by the moisture content of the lower canopy and litter layer. |
| Dimitrakopoulos, 2001 | 24 woody species; Mediterranean Basin | Leaves | Ignitability |  |  |  |  |  | - | Most of the variation in the time-to-ignition was explained by moisture content. |
| Fletcher et al., 2007 | 8 shrub species native to chaparral; California and Utah | Leaves | Ignitability |  |  |  |  |  | 0 | No consistent correlations between moisture content and the ignition behaviour of shrub leaves. |
| Murray et al., 2013 | 52 native and 27 alien species from dry sclerophyll forest, New South Wales, Australia | Dry leaves vs  fresh leaves | Ignitability | + |  |  |  |  | - | Time to ignition of fresh leaves did not differ between native and alien plants, but time to ignition of dried leaves was faster in alien than in native plants.  Leaf size (but not leaf thickness) enhanced ignitability. |
| Santacruz-Garcia et al., 2019 | 11 tree or shrub species from Chaco forests; Argentina | Plant shoots | Flammability index | + |  |  | - |  | - | Low plant height, fine branching, dense canopy and evergreen leaves with low moisture content resulted in highest flammability. |
| Santana and Marrs, 2014 | 4 genera of shrubs, and *Sphagnum* mosses from heathlands and moorlands; Britain | Leaves and plant shoots | Ignitability  Combustibility Sustainability  Consumability |  |  |  |  |  | -  -  -  - | All measures of flammability in reconstructed litter beds and reconstructed plant shoot stands were negatively affected by fuel moisture. |
| Saura-Mas et al., 2010 | 29 species from woodlands and coastal shrublands; Mediterranean Basin | Leaves | Ignitability  Combustibility | +  + |  | + |  | +  + | -  - | Direct links between flammability and fuel traits not studied.  Compared to non-seeders, reseeder species showed lower fuel loads but with higher proportion of fine, dead and dry material, leading to increased ignitability and combustibility. |
| Schwilk, 2003 | One chaparral shrub species; California | Plots  (4m x 6m) | Burn intensity | + |  | + |  | + |  | Canopy flammability (burn intensity) was significantly affected by manipulations of shrub canopy architecture. |
| Simpson et al., 2016 | 25 grass species (incl. 1 alien) from Grassland and Nama-Karoo; South Africa | Plant shoots (traits)  Leaves (flammability) | Ignitability  Combustibility Sustainability |  |  | -  +  + | +  - |  | -  -  + | Fuel biomass and fuel moisture content strongly influenced all components of flammability. |
| Wyse et al., 2016 | 60 indigenous or exotic tree or shrub species; New Zealand | Plant shoots | Flammability |  |  |  |  |  | - | The focus of the study was on comparing flammability of species, rather than linking flammability to fuel traits. |
| Wyse et al., 2018 | 6 indigenous and 4 invasive woody species of shrublands; New Zealand | Plant shoots | Ignitability  Maximum temperature  Sustainability  Consumability |  |  | + | +  - |  | -  -  - | The study compared flammability of species mixes, and the main result was that the most flammable species drives the net flammability of the species mix. |
