# Supplementary material for: Fuel trait effects on flammability of native and invasive alien shrubs in coastal fynbos and thicket (Cape Floristic Region)
Source: PeerJ. 2022 Jul 28;10:e13765. doi: 10.7717/peerj.13765 (PMC9339215; doi:10.7717/peerj.13765)
Supplement: Supplemental Information 2 — Stepwise model selection was based on the lowest Akaike information criterion (AIC). Fixed factors included in these models were fine fuels (F), coarse fuels (C), dry biomass (B), fuel bed porosity (P), dead fuels (D), and fuel moisture (M). [file peerj-10-13765-s002.docx]

**SUPPLEMENTARY 2**

Results of multiple regression models ran on the response variables maximum temperature, completeness of burn, and time-to-ignition using stepwise model selection based on the lowest Akaike information criterion (AIC). Fixed factors included in these models were fine fuels (F), coarse fuels (C), dry biomass (B), fuel bed porosity (P), dead fuels (D), and fuel moisture (M).

| Response variable | Fixed factors | AIC |
| --- | --- | --- |
| Maximum temperature | C + B + M | -18.89 |
| Maximum temperature | C + B + P + M | -18.21 |
| Maximum temperature | C + B + P + D + M | -16.50 |
| Maximum temperature | F + C + B + P + D + M | -14.54 |
| Completeness of burn | F + B + M | -5.82 |
| Completeness of burn | F + B + P + M | -5.75 |
| Completeness of burn | F + B + P + D + M | -4.05 |
| Completeness of burn | F + C + B + P + D + M | -2.68 |
| Time-to-ignition | F + M | -4.34 |
| Time-to-ignition | F + D + M | -3.69 |
| Time-to-ignition | F + B + D + M | -2.54 |
| Time-to-ignition | F + B + P + D + M | -0.88 |
| Time-to-ignition | F + C + B + P + D + M | 0.72 |
