# Supplementary material for: Fuel trait effects on flammability of native and invasive alien shrubs in coastal fynbos and thicket (Cape Floristic Region)
Source: PeerJ. 2022 Jul 28;10:e13765. doi: 10.7717/peerj.13765 (PMC9339215; doi:10.7717/peerj.13765)
Supplement: Supplemental Information 3 — Fixed factors included in these models were fine fuels, coarse fuels, dry biomass, fuel bed porosity, dead fuels, and fuel moisture. Scaled estimates were derived from incorporating the scale function in the multiple regression models. [file peerj-10-13765-s003.pdf]

Full-MultiReg-models_Fueltraits.R

samsp

2022-05-17

```
#Msweli et al_ms R1_20220510



setwd("C:/Users/samsp/Documents/1_to be published/Fueltraits/Msweli_Fueltraits paper/Fuel tra
its manuscript/")

library(readxl)
FueltraitsDataset <- read_excel("CompleteFlammability datasets_Msweli_ST - Copy.xlsx", sheet
 = "FuelTraits_Dataset")
View(FueltraitsDataset)

library(Matrix)
library(lme4)

library(car)
library(effects)

library(ggplot2)

library(knitr)
library(rmarkdown)




##STEPWISE MULTIREG MODELS

#1. Run stepwise Multiple Linear Regression -lm() Function - model for each flammability vari
able

maximumtemperatureRegM <- lm(scale(AverageMaximumTemperature) ~
                               scale(AverageFineFuels) + scale(AverageCoarseFuels) + scale(Av
erageDeadFuel)+
                               scale(AverageFBP) + scale(AverageDryBiomass) + scale(AverageFu
elMoisture),
                             data = FueltraitsDataset)
summary(maximumtemperatureRegM)
```

```
##
## Call:
## lm(formula = scale(AverageMaximumTemperature) ~ scale(AverageFineFuels) +
##     scale(AverageCoarseFuels) + scale(AverageDeadFuel) + scale(AverageFBP) +
##     scale(AverageDryBiomass) + scale(AverageFuelMoisture), data = FueltraitsDataset)
##
## Residuals:
##      Min       1Q   Median       3Q      Max
## -1.56828 -0.23074  0.00387  0.36478  1.41254
##
## Coefficients:
##                             Estimate Std. Error t value Pr(>|t|)
## (Intercept)               -2.947e-17  1.296e-01   0.000  1.00000
## scale(AverageFineFuels)    -3.490e-02  1.860e-01  -0.188  0.85279
## scale(AverageCoarseFuels)  -1.879e-01  2.557e-01  -0.735  0.46985
## scale(AverageDeadFuel)      8.095e-02  1.702e-01   0.476  0.63885
## scale(AverageFBP)           1.969e-01  1.763e-01   1.117  0.27562
## scale(AverageDryBiomass)    7.083e-01  1.910e-01   3.708  0.00116 **
## scale(AverageFuelMoisture) -4.038e-01  1.625e-01  -2.485  0.02068 *
## ---
## Signif. codes:  0 '***' 0.001 '**' 0.01 '*' 0.05 '.' 0.1 ' ' 1
##
## Residual standard error: 0.7097 on 23 degrees of freedom
## Multiple R-squared:  0.6005, Adjusted R-squared:  0.4963
## F-statistic: 5.762 on 6 and 23 DF,  p-value: 0.0008845
```

```
step(maximumtemperatureRegM, direction = "backward")
```

```
## Start:  AIC=-14.54
## scale(AverageMaximumTemperature) ~ scale(AverageFineFuels) +
##     scale(AverageCoarseFuels) + scale(AverageDeadFuel) + scale(AverageFBP) +
##     scale(AverageDryBiomass) + scale(AverageFuelMoisture)
##
##                               Df Sum of Sq    RSS      AIC
## - scale(AverageFineFuels)      1    0.0177 11.603 -16.4973
## - scale(AverageDeadFuel)       1    0.1139 11.700 -16.2496
## - scale(AverageCoarseFuels)    1    0.2720 11.858 -15.8469
## - scale(AverageFBP)            1    0.6282 12.214 -14.9590
## <none>                                     11.585 -14.5432
## - scale(AverageFuelMoisture)   1    3.1098 14.695  -9.4100
## - scale(AverageDryBiomass)     1    6.9240 18.509  -2.4874
##
## Step:  AIC=-16.5
## scale(AverageMaximumTemperature) ~ scale(AverageCoarseFuels) +
##     scale(AverageDeadFuel) + scale(AverageFBP) + scale(AverageDryBiomass) +
##     scale(AverageFuelMoisture)
##
##                               Df Sum of Sq    RSS      AIC
## - scale(AverageDeadFuel)       1    0.1126 11.716 -18.2076
## - scale(AverageCoarseFuels)    1    0.2545 11.858 -17.8465
## - scale(AverageFBP)            1    0.6123 12.216 -16.9544
## <none>                                     11.603 -16.4973
## - scale(AverageFuelMoisture)   1    3.1892 14.793 -11.2124
## - scale(AverageDryBiomass)     1    7.4841 19.087  -3.5651
##
## Step:  AIC=-18.21
## scale(AverageMaximumTemperature) ~ scale(AverageCoarseFuels) +
##     scale(AverageFBP) + scale(AverageDryBiomass) + scale(AverageFuelMoisture)
##
##                               Df Sum of Sq    RSS      AIC
## - scale(AverageFBP)            1    0.5254 12.241 -18.8915
## - scale(AverageCoarseFuels)    1    0.6493 12.365 -18.5894
## <none>                                     11.716 -18.2076
## - scale(AverageFuelMoisture)   1    3.0968 14.813 -13.1715
## - scale(AverageDryBiomass)     1    7.4689 19.185  -5.4125
##
## Step:  AIC=-18.89
## scale(AverageMaximumTemperature) ~ scale(AverageCoarseFuels) +
##     scale(AverageDryBiomass) + scale(AverageFuelMoisture)
##
##                               Df Sum of Sq    RSS      AIC
## <none>                                     12.241 -18.8915
## - scale(AverageCoarseFuels)    1    1.4268 13.668 -17.5840
## - scale(AverageFuelMoisture)   1    2.7869 15.028 -14.7380
## - scale(AverageDryBiomass)     1    6.9701 19.211  -7.3709
```

```
##
## Call:
## lm(formula = scale(AverageMaximumTemperature) ~ scale(AverageCoarseFuels) +
##     scale(AverageDryBiomass) + scale(AverageFuelMoisture), data = FueltraitsDataset)
##
## Coefficients:
##                (Intercept)   scale(AverageCoarseFuels)    scale(AverageDryBiomass)
##                 -2.447e-17                  -3.209e-01                   6.765e-01
## scale(AverageFuelMoisture)
##                 -3.640e-01
```

```
COBRegM <- lm(scale(AverageCompletenessOfBurn) ~
                scale(AverageFineFuels) + scale(AverageCoarseFuels) + scale(AverageDeadFuel)+
                scale(AverageFBP) + scale(AverageDryBiomass) + scale(AverageFuelMoisture),
              data = FueltraitsDataset)
summary(COBRegM)
```

```
##
## Call:
## lm(formula = scale(AverageCompletenessOfBurn) ~ scale(AverageFineFuels) +
##     scale(AverageCoarseFuels) + scale(AverageDeadFuel) + scale(AverageFBP) +
##     scale(AverageDryBiomass) + scale(AverageFuelMoisture), data = FueltraitsDataset)
##
## Residuals:
##      Min       1Q   Median       3Q      Max
## -1.70281 -0.49944  0.07098  0.58951  1.32703
##
## Coefficients:
##                             Estimate Std. Error t value Pr(>|t|)
## (Intercept)               -9.183e-16  1.579e-01   0.000   1.0000
## scale(AverageFineFuels)     3.590e-01  2.266e-01   1.584   0.1269
## scale(AverageCoarseFuels)  -2.185e-01  3.117e-01  -0.701   0.4902
## scale(AverageDeadFuel)     -1.598e-01  2.074e-01  -0.771   0.4488
## scale(AverageFBP)           1.835e-01  2.148e-01   0.854   0.4018
## scale(AverageDryBiomass)    4.965e-01  2.328e-01   2.132   0.0439 *
## scale(AverageFuelMoisture) -2.348e-01  1.980e-01  -1.186   0.2479
## ---
## Signif. codes:  0 '***' 0.001 '**' 0.01 '*' 0.05 '.' 0.1 ' ' 1
##
## Residual standard error: 0.8649 on 23 degrees of freedom
## Multiple R-squared:  0.4067, Adjusted R-squared:  0.252
## F-statistic: 2.628 on 6 and 23 DF,  p-value: 0.04337
```

```
step(COBRegM, direction = "backward")
```

```
## Start:  AIC=-2.68
## scale(AverageCompletenessOfBurn) ~ scale(AverageFineFuels) +
##     scale(AverageCoarseFuels) + scale(AverageDeadFuel) + scale(AverageFBP) +
##     scale(AverageDryBiomass) + scale(AverageFuelMoisture)
##
##                              Df Sum of Sq    RSS     AIC
## - scale(AverageCoarseFuels)   1    0.3678 17.573 -4.0454
## - scale(AverageDeadFuel)      1    0.4441 17.649 -3.9154
## - scale(AverageFBP)           1    0.5459 17.751 -3.7428
## - scale(AverageFuelMoisture)  1    1.0516 18.256 -2.9003
## <none>                                    17.205 -2.6800
## - scale(AverageFineFuels)     1    1.8765 19.081 -1.5744
## - scale(AverageDryBiomass)    1    3.4017 20.607  0.7325
##
## Step:  AIC=-4.05
## scale(AverageCompletenessOfBurn) ~ scale(AverageFineFuels) +
##     scale(AverageDeadFuel) + scale(AverageFBP) + scale(AverageDryBiomass) +
##     scale(AverageFuelMoisture)
##
##                              Df Sum of Sq    RSS     AIC
## - scale(AverageDeadFuel)      1   0.17392 17.747 -5.7499
## - scale(AverageFBP)           1   1.14236 18.715 -4.1559
## <none>                                    17.573 -4.0454
## - scale(AverageFuelMoisture)  1   1.89459 19.467 -2.9737
## - scale(AverageFineFuels)     1   2.57437 20.147 -1.9440
## - scale(AverageDryBiomass)    1   3.03554 20.608 -1.2651
##
## Step:  AIC=-5.75
## scale(AverageCompletenessOfBurn) ~ scale(AverageFineFuels) +
##     scale(AverageFBP) + scale(AverageDryBiomass) + scale(AverageFuelMoisture)
##
##                              Df Sum of Sq    RSS     AIC
## - scale(AverageFBP)           1    1.1762 18.923 -5.8247
## <none>                                    17.747 -5.7499
## - scale(AverageFuelMoisture)  1    1.7893 19.536 -4.8682
## - scale(AverageFineFuels)     1    2.4136 20.160 -3.9245
## - scale(AverageDryBiomass)    1    3.5504 21.297 -2.2788
##
## Step:  AIC=-5.82
## scale(AverageCompletenessOfBurn) ~ scale(AverageFineFuels) +
##     scale(AverageDryBiomass) + scale(AverageFuelMoisture)
##
##                              Df Sum of Sq    RSS     AIC
## <none>                                    18.923 -5.8247
## - scale(AverageFuelMoisture)  1    1.6594 20.582 -5.3029
## - scale(AverageDryBiomass)    1    2.5612 21.484 -4.0164
## - scale(AverageFineFuels)     1    3.5784 22.501 -2.6286
```

```
##
## Call:
## lm(formula = scale(AverageCompletenessOfBurn) ~ scale(AverageFineFuels) +
##     scale(AverageDryBiomass) + scale(AverageFuelMoisture), data = FueltraitsDataset)
##
## Coefficients:
##                (Intercept)     scale(AverageFineFuels)    scale(AverageDryBiomass)
##                 -8.967e-16                   4.518e-01                   3.666e-01
## scale(AverageFuelMoisture)
##                 -2.690e-01
```

```
TimeToIgnitionRegM <- lm(scale(AverageTimeToIgnition) ~
                           scale(AverageFineFuels) + scale(AverageCoarseFuels) + scale(Averag
eDeadFuel)+
                           scale(AverageFBP) + scale(AverageDryBiomass) + scale(AverageFuelMo
isture),
                         data = FueltraitsDataset)
summary(TimeToIgnitionRegM)
```

```
##
## Call:
## lm(formula = scale(AverageTimeToIgnition) ~ scale(AverageFineFuels) +
##     scale(AverageCoarseFuels) + scale(AverageDeadFuel) + scale(AverageFBP) +
##     scale(AverageDryBiomass) + scale(AverageFuelMoisture), data = FueltraitsDataset)
##
## Residuals:
##      Min       1Q   Median       3Q      Max
## -1.85758 -0.51614 -0.01551  0.50059  2.23063
##
## Coefficients:
##                             Estimate Std. Error t value Pr(>|t|)
## (Intercept)               -3.319e-17  1.671e-01   0.000    1.000
## scale(AverageFineFuels)   -3.089e-01  2.398e-01  -1.288    0.211
## scale(AverageCoarseFuels)  1.834e-01  3.298e-01   0.556    0.584
## scale(AverageDeadFuel)     2.953e-01  2.195e-01   1.345    0.192
## scale(AverageFBP)          1.581e-01  2.273e-01   0.695    0.494
## scale(AverageDryBiomass)   1.725e-01  2.464e-01   0.700    0.491
## scale(AverageFuelMoisture) 3.166e-01  2.096e-01   1.511    0.144
##
## Residual standard error: 0.9153 on 23 degrees of freedom
## Multiple R-squared:  0.3356, Adjusted R-squared:  0.1623
## F-statistic: 1.936 on 6 and 23 DF,  p-value: 0.1176
```

```
step(TimeToIgnitionRegM, direction = "backward")
```

```
## Start:  AIC=0.72
## scale(AverageTimeToIgnition) ~ scale(AverageFineFuels) + scale(AverageCoarseFuels) +
##     scale(AverageDeadFuel) + scale(AverageFBP) + scale(AverageDryBiomass) +
##     scale(AverageFuelMoisture)
##
##                              Df Sum of Sq    RSS      AIC
## - scale(AverageCoarseFuels)   1   0.25907 19.527 -0.88169
## - scale(AverageFBP)           1   0.40514 19.673 -0.65813
## - scale(AverageDryBiomass)    1   0.41075 19.679 -0.64956
## <none>                                    19.268  0.71763
## - scale(AverageFineFuels)     1   1.38972 20.658  0.80691
## - scale(AverageDeadFuel)      1   1.51611 20.784  0.98991
## - scale(AverageFuelMoisture)  1   1.91227 21.180  1.55634
##
## Step:  AIC=-0.88
## scale(AverageTimeToIgnition) ~ scale(AverageFineFuels) + scale(AverageDeadFuel) +
##     scale(AverageFBP) + scale(AverageDryBiomass) + scale(AverageFuelMoisture)
##
##                              Df Sum of Sq    RSS      AIC
## - scale(AverageFBP)           1   0.22366 19.751 -2.54004
## - scale(AverageDryBiomass)    1   0.76417 20.291 -1.73007
## - scale(AverageDeadFuel)      1   1.27434 20.802 -0.98514
## <none>                                    19.527 -0.88169
## - scale(AverageFineFuels)     1   1.89612 21.423 -0.10154
## - scale(AverageFuelMoisture)  1   2.97053 22.498  1.36649
##
## Step:  AIC=-2.54
## scale(AverageTimeToIgnition) ~ scale(AverageFineFuels) + scale(AverageDeadFuel) +
##     scale(AverageDryBiomass) + scale(AverageFuelMoisture)
##
##                              Df Sum of Sq    RSS     AIC
## - scale(AverageDryBiomass)    1   0.56922 20.320 -3.6877
## - scale(AverageDeadFuel)      1   1.23775 20.989 -2.7165
## <none>                                    19.751 -2.5400
## - scale(AverageFineFuels)     1   1.68783 21.439 -2.0800
## - scale(AverageFuelMoisture)  1   3.04566 22.797 -0.2377
##
## Step:  AIC=-3.69
## scale(AverageTimeToIgnition) ~ scale(AverageFineFuels) + scale(AverageDeadFuel) +
##     scale(AverageFuelMoisture)
##
##                              Df Sum of Sq    RSS     AIC
## - scale(AverageDeadFuel)      1    0.9339 21.254 -4.3395
## <none>                                    20.320 -3.6877
## - scale(AverageFuelMoisture)  1    2.4913 22.811 -2.2182
## - scale(AverageFineFuels)     1    3.8132 24.133 -0.5282
##
## Step:  AIC=-4.34
## scale(AverageTimeToIgnition) ~ scale(AverageFineFuels) + scale(AverageFuelMoisture)
##
##                              Df Sum of Sq    RSS     AIC
## <none>                                    21.254 -4.3395
## - scale(AverageFuelMoisture)  1    2.3872 23.641 -3.1462
## - scale(AverageFineFuels)     1    2.9586 24.213 -2.4297
```

```
##
## Call:
## lm(formula = scale(AverageTimeToIgnition) ~ scale(AverageFineFuels) +
##     scale(AverageFuelMoisture), data = FueltraitsDataset)
##
## Coefficients:
##                (Intercept)     scale(AverageFineFuels)  scale(AverageFuelMoisture)
##                 -1.207e-18                  -3.361e-01                   3.019e-01
```

```
#1.2 run the MultiReg models with the lowest AIC

MaximumtemperatureMod <- lm(formula = scale(AverageMaximumTemperature) ~ scale(AverageCoarseF
uels) +
                              scale(AverageDryBiomass) + scale(AverageFuelMoisture),
                            data = FueltraitsDataset)
summary(MaximumtemperatureMod)
```

```
##
## Call:
## lm(formula = scale(AverageMaximumTemperature) ~ scale(AverageCoarseFuels) +
##     scale(AverageDryBiomass) + scale(AverageFuelMoisture), data = FueltraitsDataset)
##
## Residuals:
##      Min       1Q   Median       3Q      Max
## -1.69005 -0.29667 -0.02941  0.36152  1.20850
##
## Coefficients:
##                              Estimate Std. Error t value Pr(>|t|)
## (Intercept)                -2.447e-17  1.253e-01   0.000 1.000000
## scale(AverageCoarseFuels)  -3.209e-01  1.843e-01  -1.741 0.093535 .
## scale(AverageDryBiomass)    6.765e-01  1.758e-01   3.848 0.000695 ***
## scale(AverageFuelMoisture) -3.640e-01  1.496e-01  -2.433 0.022159 *
## ---
## Signif. codes:  0 '***' 0.001 '**' 0.01 '*' 0.05 '.' 0.1 ' ' 1
##
## Residual standard error: 0.6862 on 26 degrees of freedom
## Multiple R-squared:  0.5779, Adjusted R-squared:  0.5292
## F-statistic: 11.86 on 3 and 26 DF,  p-value: 4.406e-05
```

```
COBMod <- lm(formula = scale(AverageCompletenessOfBurn) ~ scale(AverageFineFuels) +
               scale(AverageDryBiomass) + scale(AverageFuelMoisture),
             data = FueltraitsDataset)
summary(COBMod)
```

```
##
## Call:
## lm(formula = scale(AverageCompletenessOfBurn) ~ scale(AverageFineFuels) +
##     scale(AverageDryBiomass) + scale(AverageFuelMoisture), data = FueltraitsDataset)
##
## Residuals:
##      Min       1Q   Median       3Q      Max
## -1.76016 -0.53417  0.00984  0.60709  1.45938
##
## Coefficients:
##                              Estimate Std. Error t value Pr(>|t|)
## (Intercept)                -8.967e-16  1.558e-01   0.000   1.0000
## scale(AverageFineFuels)     4.518e-01  2.037e-01   2.217   0.0355 *
## scale(AverageDryBiomass)    3.666e-01  1.954e-01   1.876   0.0719 .
## scale(AverageFuelMoisture) -2.690e-01  1.781e-01  -1.510   0.1431
## ---
## Signif. codes:  0 '***' 0.001 '**' 0.01 '*' 0.05 '.' 0.1 ' ' 1
##
## Residual standard error: 0.8531 on 26 degrees of freedom
## Multiple R-squared:  0.3475, Adjusted R-squared:  0.2722
## F-statistic: 4.615 on 3 and 26 DF,  p-value: 0.0102
```

```
TimeToIgnitionMod <- lm(formula = scale(AverageTimeToIgnition) ~ scale(AverageFineFuels) + sc
ale(AverageFuelMoisture),
                        data = FueltraitsDataset)
summary(TimeToIgnitionMod)
```

```
##
## Call:
## lm(formula = scale(AverageTimeToIgnition) ~ scale(AverageFineFuels) +
##     scale(AverageFuelMoisture), data = FueltraitsDataset)
##
## Residuals:
##      Min       1Q   Median       3Q      Max
## -1.82846 -0.43221  0.02414  0.33697  1.96583
##
## Coefficients:
##                              Estimate Std. Error t value Pr(>|t|)
## (Intercept)                -1.207e-18  1.620e-01   0.000   1.0000
## scale(AverageFineFuels)    -3.361e-01  1.733e-01  -1.939   0.0631 .
## scale(AverageFuelMoisture)  3.019e-01  1.733e-01   1.741   0.0930 .
## ---
## Signif. codes:  0 '***' 0.001 '**' 0.01 '*' 0.05 '.' 0.1 ' ' 1
##
## Residual standard error: 0.8872 on 27 degrees of freedom
## Multiple R-squared:  0.2671, Adjusted R-squared:  0.2128
## F-statistic:  4.92 on 2 and 27 DF,  p-value: 0.01507
```
