# Supplementary material for: Fuel trait effects on flammability of native and invasive alien shrubs in coastal fynbos and thicket (Cape Floristic Region)
Source: PeerJ. 2022 Jul 28;10:e13765. doi: 10.7717/peerj.13765 (PMC9339215; doi:10.7717/peerj.13765)
Supplement: Supplemental Information 4 [file peerj-10-13765-s004.docx]

**SUPPLEMENTARY 4**

Kruskal Wallis results of the comparison of flammability measures and fuel traits (respectively) among species from the vegetation groups, IAPs, fynbos, and thicket.

| Factors | H_(2)_ | p |
| --- | --- | --- |
| Maximum temperature | 4.50 | 0.11 |
| Completeness of burn | 6.29 | 0.04 |
| Time-to-ignition | 13.43 | 0.00 |
| Fine fuels | 13.52 | 0.00 |
| Coarse fuels | 14.89 | 0.00 |
| Dead fuels | 5.07 | 0.08 |
| Fuel bed porosity | 6.20 | 0.04 |
| Dry biomass | 8.30 | 0.02 |
| Fuel moisture | 3.38 | 0.18 |
